# MDA19, a novel CB2 agonist, inhibits hepatocellular carcinoma partly through inactivation of AKT signaling pathway

**DOI:** 10.1186/s13062-019-0241-1

**Published:** 2019-05-03

**Authors:** Mei Rao, Dongfeng Chen, Peng Zhan, Jianqing Jiang

**Affiliations:** 1 Department of Pharmacy, Longyan First Hospital Affiliated to Fujian Medical University, 105 Jiuyi North Road, Longyan, Fujian 364000 People’s Republic of China; 2 Department of Osteology, Longyan First Hospital Affiliated to Fujian Medical University, 105 Jiuyi North Road, Longyan, 364000 Fujian People’s Republic of China

**Keywords:** MDA19, CB_2_, HCC, Apoptosis, AKT signaling pathway

## Abstract

**Background:**

CB_2_ (cannabinoid receptor 2) agonists have been shown to exert anti-tumor activities in different tumor types. However, there is no study exploring the role of MDA19 (a novel CB_2_ agonist) in tumors. In this study we aimed to investigate the effects of MDA19 treatment on HCC cell lines, Hep3B and HepG2 and determine the relevant mechanisms.

**Results:**

Cell proliferation analysis, including CCK8 and colony formation assays, indicated that MDA19 treatment inhibited HCC cell proliferation in a dose- and time-dependent manner. Flow cytometry suggested that MDA19 induced cell apoptosis and activation of mitochondrial apoptosis pathway. Transwell assay indicated that HCC cell migration and invasion were significantly inhibited by MDA19 treatment. Mechanism investigation suggested that MDA19 induced inactivation of AKT signaling pathway in HCC cells. In addition, we investigated the function of CB_2_receptor in HCC and its role in the anti-tumor activity of MDA19. By searching on Kaplan-Meier plotter (http://kmplot.com/analysis/), we found that HCC patients with high CB_2_ expression had a better survival and CB2 expression was significantly associated with gender, clinical stages and race of HCC patients (*P* < 0.05). CB_2_ inhibited the progression of HCC cells and its knockdown could rescue the growth inhibition induced by MDA19 in HCC. Moreover, the inhibitory effect of MDA19 on AKT signaling pathway was also reversed by CB_2_ knockdown.

**Conclusion:**

Our data suggest that MDA-19 exerts an anti-tumor activity at least partly through inactivation of AKT signaling pathway in HCC. CB_2_ functions as a tumor suppressor gene in HCC, and MDA19-induced growth inhibition of HCC cells depends on its binding to CB_2_ to activate it. MDA-19 treatment may be a promising strategy for HCC therapy.

**Reviewer:**

This article was reviewed by Tito Cali, Mohamed Naguib and Bo Chen.

## Background

Hepatocellular carcinoma (HCC) is one of the most common tumors worldwide and ranks as the third leading cause of cancer-related deaths, killing more than 600,000 people each year [1, 2]. Previous reports have identified many risk factors, including alcohol use, liver cirrhosis, hepatitis.

B/C virus (HBV/HCV) infection and environmental pollution [3]. Current therapies for HCC mainly include liver resection and transplantation, percutaneous ablation, and chemotherapy [1, 3]. However, the treatment status of HCC patients still remains unsatisfactory and the The five-year survival rate for HCC patients is only 3–5% [4–6]. Therefore, it is increasingly important to explore the regulatory mechanisms of HCC carcinogenesis and identify effective therapeutic targets.

In recent years it has been found that Cannabinoids exert anti-tumor activities by inducing tumor cell death, cell cycle arrest or inhibiting tumor angiogenesis [7, 8]. The function of Cannabinoids depends on its interaction with the endocannabinoid system (ECS), including CB_1_ or CB_2_. CB_1_ is mainly expressed in the central nervous system whereas CB2 is expressed in peripheral or immune tissues [9]. N′-[(3Z)-1-(1-hexyl)-2-oxo-1, 2-dihydro-3H-indol-3-ylidene] benzohydrazide (MDA19), as a selective agonist for CB_1_ and CB_2_, has been demonstrated to play an important role in relieving neuropathic pain without a potential adverse effect on the central nervous system [10]. According to a previous study, MDA19 displayed 4-fold-higher affinity at the human CB_2_ than at the human CB_1_ receptor and nearly 70-fold-higher affinity at the rat CB_2_ than at the rat CB_1_ receptor [11]. At present, some agonists of CB_2_ or CB_1_ have been shown to exert anti-tumor activity in some tumor models [12–14]. However, there is currently no study on the role of MDA19 in tumors.

In this study, we investigated the effects of MDA19 on tumor-related phenotypes in Hep3B and HepG2 HCC cells. AKT signaling pathway was investigated to explain the action mechanism of MDA19 in HCC. In addition, we investigated the prognostic value of CB_2_ in HCC and its correlation with clinical pathological parameters of patients. The functions of CB_2_ in HCC were also investigated by using siRNA technology. Finally, we evaluated the role of CB_2_ in the anti-tumor activity of MDA19 in HCC.

## Methods

### Cells culture and treatment

HCC cell lines, Hep3B and HepG2, were purchased from the Cell Bank of the Chinese Academy of Sciences (Shanghai, China), and cultured in DMEM (HyClone, Logan, UT) medium supplemented with 10% fetal bovine serum (FBS, GibcoBRL; Grand Island, NY, USA) at 37 °C with 5% CO_2_. MDA19 (Cat# HY-15451, MedChemExpress, USA) was dissolved in DMSO and then diluted with DMEM to a specific concentration to incubate with HCC cells. siRNA targeting CB_2_ and a negative control siRNA (siNC) were synthesized by Guangzhou RiboBio Co., Ltd. and transfected into HCC cells by using Lipofectamine2000 liposomes (Invitrogen; Thermo Fisher Scientific, Inc., Waltham, MA, USA). The sequences of siRNAs were as follows:

CB_2_ siRNA1: 5′-CCAGGTCAAGAAGGCCTTT-3′;

CB_2_ siRNA2: 5′- GCTTGGATTCCAACCCTAT-3′;

CB_2_ siRNA3: 5′-CCTGGCCAGTGTGGTCTTT-3′;

siNC: 5′-UUCUCCGAACGUGUCACGUTT-3.

### Quantitative real-time reverse transcriptase-PCR (qRT-PCR) assay

After transfection with CB_2_ siRNA or siNC for 48 h, total RNA from HCC cells was isolated using TRIzol (Invitrogen, Carlsbad, CA, USA) according to the manufacturer’s instructions. 1 μg of RNA was used for cDNA synthesis by a RT-reaction using using the RevertAid First Strand cDNA Synthesis kit (Thermo Fisher Scientific, Shanghai, China) according to the manufacturer’s instructions. The PCR reaction was performed using Mx3005P Real-Time PCR Cycler (Stratagene Corp.; Agilent Technologies, Inc., USA) using SYBR-Green PCR Master Mix (Promega, Madison, WI, USA) with β-actin as an internal control. The mRNA expression of CB_2_ was quantified using the 2^-ΔΔCt^method. The used primers were as follows:

CB_2_-F: 5′-GGCTGTGCTCCTCATCTGTT-3′,

CB_2_-R: 5′-AGGATCTCGGGGCTTCTTCT-3′;

β-actin-F: 5′-GCATGGGTCAGAAGGATTCCT-3′,

β-actin-R: 5′-TCGTCCCAGTTGGTGACGAT-3′.

### Dose-dependent assay

Hep3B and HepG2 cells were treated with MDA19 of a concentration gradient (0, 5, 10, 20, 30, 40, 50, 80, 100, and 120 μM) for 48 h. Cell proliferation was analyzed by using CCK8 kit (Beijing Solarbio Science & Technology, Beijing, China).

### Cell proliferation assay

CCK8 assay: HCC cells were planted into a 96-well plate at nearly 3000 per well. After treatment with MDA19 or siRNA transfection, 10 μl CCK8 solution was added to each well at 0 h, 24 h, 48 h, 72 h and incubated for 2 h. Cell viability was quantified by measuring OD values at 450 nm using a microplate reader. The proliferation curves were plotted.

Clone formation assay: HCC cells were treated with MDA19 or siRNA transfection for 48 h. Then cells were collected and seeded in a 6-cm dish at a total number of 200~300. Cells were continually cultured until clones could be observed in naked eyes. After stained with 0.1% crystal violet for 20 min and washed with PBS twice, HCC cells were imaged and counted.

### Migration and invasion analysisBDI90241

For invasion assay, Matrigel-coated transwell inserts (BD Biosciences, San Jose, CA, USA) were prepared. Then, HCC cells treated with MDA19 or siRNA transfection were seeded into the upper chambers at a number of 1 × 10^4^ and the lower chambers were added with serum-free medium. After incubation for 48 h, non-invaded cells on the upper surface of transwell membrane were removed by a cotton swab and invaded cells were fixed with 4% paraformaldehyde for 30 min, stained with crystal violet for 30 min, and counted under a microscope. Migration assay was similar to the invasion assay except that transwell chambers weren’t pre-coated with Matrigel and the number of seeded cells was 5 × 10^3^.

### Apoptosis analysis

Cell apoptosis was analyzed by a typical PI-AnnexinV-FITC assay. Briefly, after treated with MDA19 for 48 h or siRNA transfection for 48 h, HCC cells were starved in serum-free medium for 24 h. The cells in control group were also starved in serum-free medium for 24 h. After that, cells were washed with PBS and then mixed with PI-AnnexinV-FITC for 15 min in the dark. Cell apoptosis was detected by a flow cytometer (BD FACSC anto II, BD Biosciences, USA). The data were analyzed using FlowJo software (Tree Star).

### Western blot assay

Total proteins of cells were extracted by using ice-cold RIPA Lysis Buffer (CWBIO, Beijing, China) added with 1% protease inhibitor. Protein concentrations were determined by using BCA Protein Assay Kit (Beyotime, China). Equal amounts of proteins (20 μg) from each sample were subjected to a 10% SDS-PAGE electrophoresis and transferred onto a PVDF membrane (Millipore, Bedford, USA). After blocking in 5% non-fat milk for 1 h, protein bands were successively incubated with primary antibodies at 4 °C overnight and secondary antibodies for 1 h at room temperature. After being washed three times, protein bands were developed by using chemiluminescence detection kit (Thermo Scientific, USA). The primary antibodies against Bcl2 (Cat# 2872, 1:1000), Bax (Cat# 2774, 1:1000), Caspase 3 (Cat# 9662, 1:1000), GAPDH (Cat# 5174, 1:10000), AKT (Cat# 9272, 1:1000), p-AKT (Cat# 4060, 1:1000), CDK4 (Cat# 12790, 1:1000), CDK6 (Cat# 13331, 1:1000) and Cyclin D1 (Cat# 2978, 1:1000) were purchased from Cell Signaling Technology (Beverly, MA, USA). All secondary antibodies were purchased from Invitrogen (Carlsbad, CA, USA).

### Statistical analysis

All statistical analysis was performed by using GraphPad Prism 7.0 software (GraphPad Software Inc., La Jolla, CA, USA). Comparison of two groups was subjected to unpaired Student’s t-test, indicated by t value. One-way ANOVA was performed to compare three or more groups followed by Turkey’s post hoc test. *P* < 0.05 was considered statistically significant.

## Results

### MDA19 inhibited proliferation of HCC cells in a dose- and time-dependent manner

**Fig. 1:**
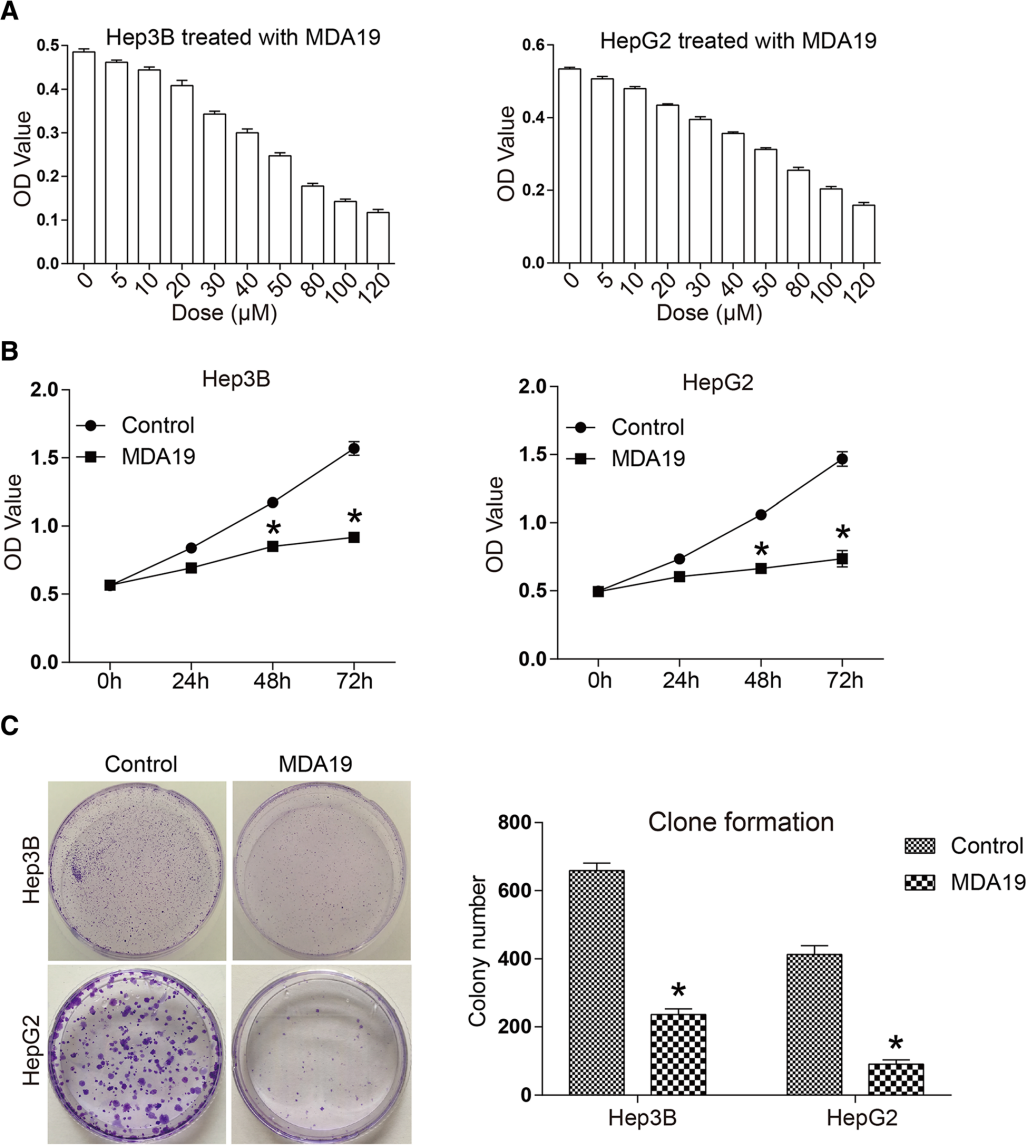
MDA19 treatment inhibited HCC cell proliferation in a dose- and time-dependent manner. **a** Hep3B (Left) and HepG2 (Right) cells were treated with a concentration gradient of MDA19 (0, 5, 10, 20, 30, 40, 50, 80, 100, 120 μM) for 48 h and then cell proliferation was detected by CCK8 assay; (**b**) Hep3B (Left) and HepG2 (Right) cells were treated with MDA19 at 30 μM and 40 μM, respectively. Cell proliferation was detected at 0 h, 24 h, 48 h and 72 h using CCK8 assays; (**c**) Hep3B and HepG2 cells were treated with MDA19 for 48 h at 30 μM and 40 μM, respectively. Then the clone formation ability of HCC cells was detected. All experiments were performed at 3 times. *P < 0.05

As shown in Fig. 1a, cell proliferation of Hep3B and HepG2 was inhibited by MDA19 in a dose-dependent manner. The significant cytotoxic effect of MDA19 was starting at concentration of 10 μM for both HCC cell lines. At 50 μM, cell viability of Hep3B and HepG2 cells was significantly inhibited by 49 and 41% respectively. The calculated IC50 values were 56.69 μM and 71.13 μM for Hep3B and HepG2 cells, respectively. 30 μM for Hep3B cells and 40 μM for HepG2 cells were used for the following experiments. The result in Fig. 1b indicated that MDA19 inhibited the proliferation of Hep3B and HepG2 cells in a time-dependent manner. At 72 h, cell viability was respectively inhibited to 58 and 50% in Hep3B and HepG2 cells. Colony formation ability of HCC cells was also significantly inhibited by MDA19. Colony number of MDA19 treatment group was decreased to 37 and 18% in Hep3B and HepG2 cells respectively (Fig. 1c). The results suggested that MDA19 inhibited HCC cell proliferation in a dose- and time-dependent manner.

### MDA19 induced apoptosis of HCC cells by activating mitochondrial apoptosis pathway

**Fig. 2:**
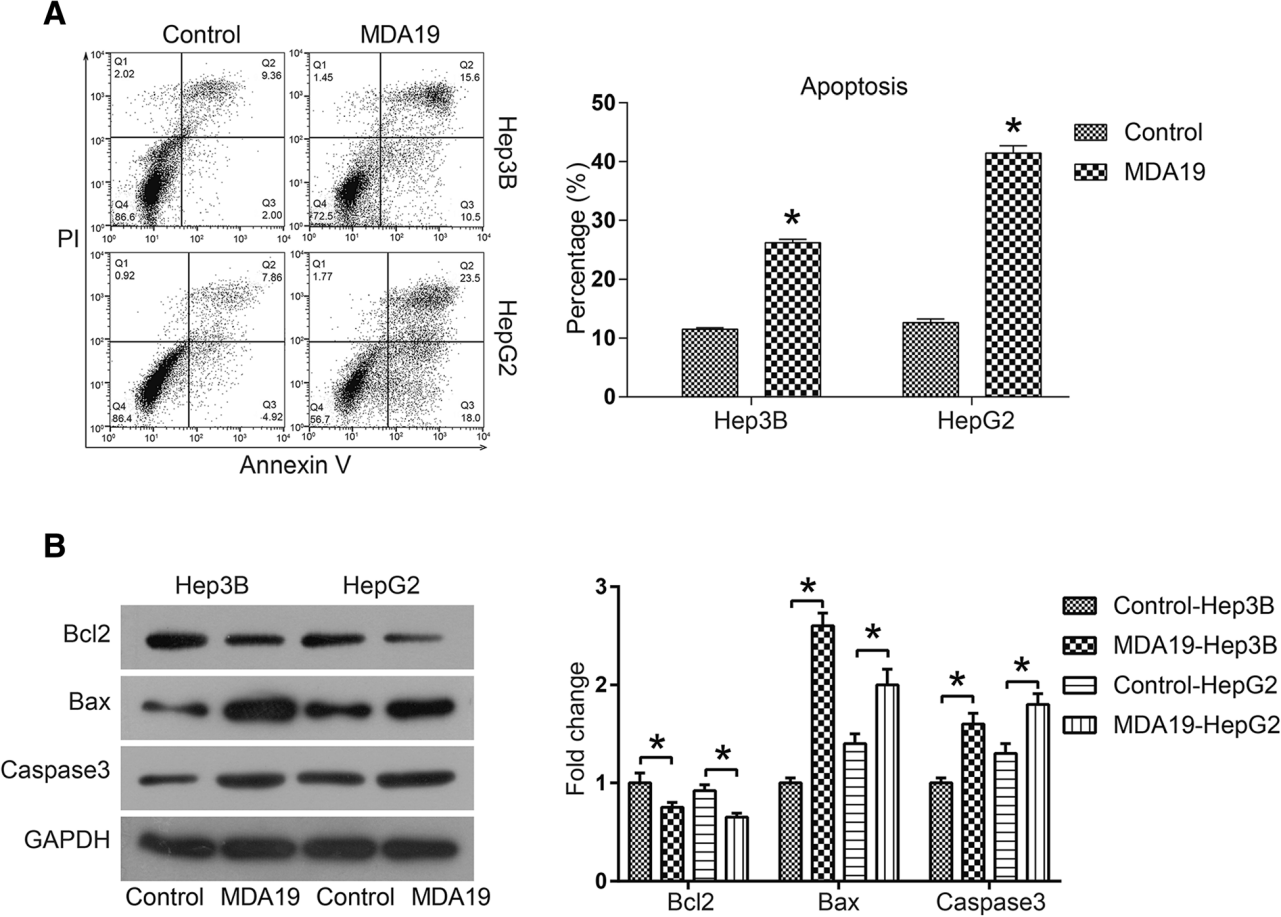
MDA19 induced apoptosis of HCC cells by activating mitochondrial-dependent apoptosis pathway. **a** Cell apoptosis of HCC cells treated with MDA19 (30 μM for Hep3B and 40 μM for HepG2) for 48 h was detected by a PI-AnnexinV-FITC assay and flow cytometry; The data were analyzed using FlowJo software. **b** Hep3B and HepG2 cells were treated with MDA19 for 48 h at 30 μM and 40 μM, respectively. Then the expression of apoptosis related proteins Bcl2, Bax and Caspase3 was detected by western blot and analyzed by Image J software. All experiments were performed at 3 times. *P < 0.05

In order to determine whether MDA19-induced growth inhibition was mediated by cell apoptosis, a PI-AnnexinV-FITC assay was performed. As shown in Fig. 2a, the percentage of apoptotic cells was increased from 11.36% of control group to 26.1% of MDA19 group for Hep3B cells and 12.78 to 41.5% for HepG2 cells. Next, we investigated whether mitochondrial apoptosis pathway was affected by MDA19 treatment. The results indicated that MDA19 treatment significantly increased the expression of pro-apoptotic protein Bax and decreased the expression of anti-apoptotic protein Bcl2 in both Hep3B and HepG2 cells (*P* < 0.05, Fig. 2b). Moreover, Caspase3, the apoptotic executioner, was also significantly down-regulated (*P* < 0.05, Fig. 2b). Taken together, these data suggested that MDA19 promoted HCC cell apoptosis by activating mitochondrial apoptosis pathway.

### MDA19 inhibited migration and invasion of HCC cells

**Fig. 3:**
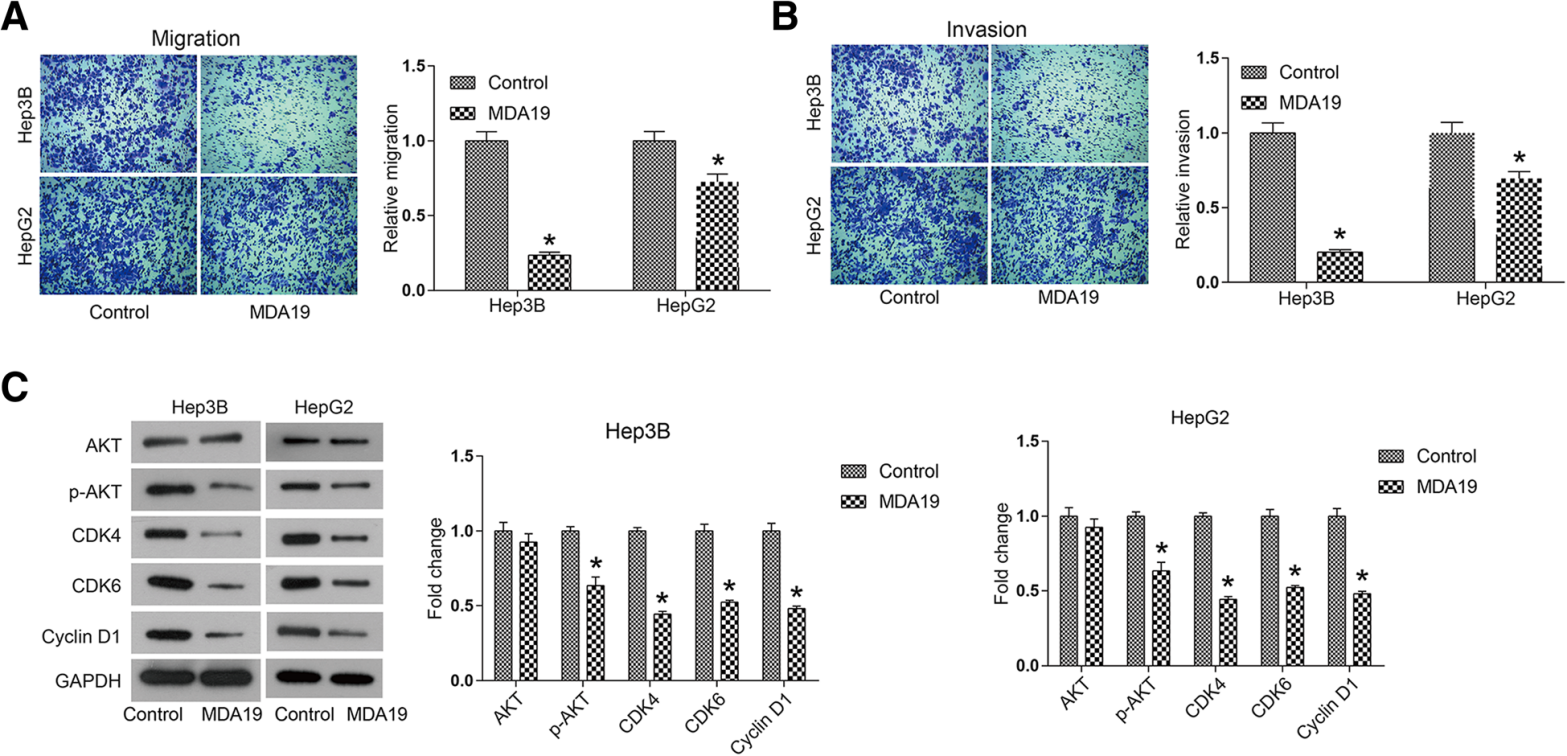
MDA19 inhibited migration and invasion and inactivated AKT signaling pathway in HCC cells. HCC cells were treated with MDA19 (30 μM for Hep3B and 40 μM for HepG2) for 48 h. (**a**) Cell migration and (**b**) cell invasion were detected by transwell assays. (C) AKT signaling pathway components, including AKT, p-AKT, CDK4, CDK6 and Cyclin D1, were detected by western blot and analyzed by Image J software. All experiments were performed at 3 times. *P < 0.05

Invasion and migration are important features of cancer cells, which frequently initiate tumor metastasis in vivo. To further investigate whether MDA19 affected cell mobility of HCC, a transwell assay was performed. As shown in Fig. 3a, HCC cell migration was significantly inhibited by MDA19, inhibitory rate reaching 76.6% for Hep3B cells and 27.5% for HepG2 cells (*P* < 0.05). Figure 3b revealed that MDA19 inhibited Hep3B cell invasion by 79.8% and HepG2 by 30.5%. Thus, it was suggested that MDA19 restrained the invasive and migratory abilities of human HCC cells.

### MDA19 inactivated AKT signaling pathway in HCC cells

We next explored the mechanism underlying the cellular phenotype alterations induced by MDA19 treatment in HCC. AKT signaling pathway is widely reported to participate in tumor cell proliferation, survival and malignant development [15–17]. As shown in Fig. 3c, phosphorylation level of AKT was significantly decreased in MDA19 treated HCC cells *(P* < 0.05). Cyclin D1 and CDK4/6 are downstream proteins of p-AKT, which form a complex to control cell cycle progression [18]. It was suggested that MDA19 treatment inhibited the expression of Cyclin D1 and CDK4/6 significantly (*P* < 0.05, Fig. 3). Taken together, these data suggested that MDA19 treatment inhibited HCC progression at least partly through inactivation of AKT signaling pathway.

### High expression of MDA19 receptor CB_2_ predicted a better prognosis in HCC

**Fig. 4:**
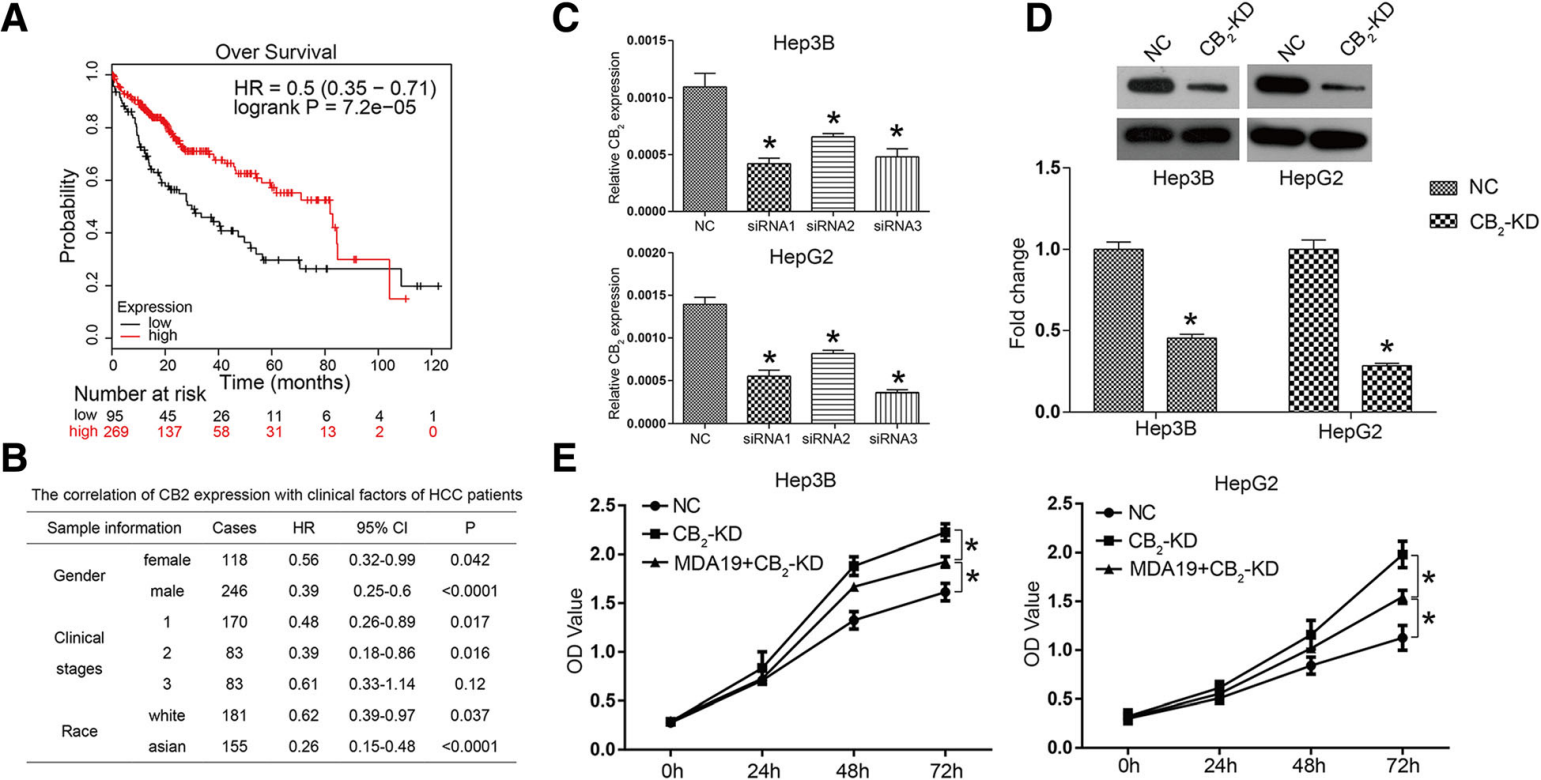
CB_2_ high expression represented a better prognosis of HCC patients and CB2 knockdown reversed the inhibition of HCC cell proliferation induced by MDA19. **a** Kaplan-Meier plotter (http://kmplot.com/analysis/) is a bioinformatics website that assesses the effect of 54,675 genes on survival using 10,461 cancer samples. The survival curves of HCC patients with high or low CB_2_ mRNA level were acquired by searching on Kaplan-Meier plotter; (Affy id/Gene symbol: 1269_s_at; Survival: RFS (*n* = 364); Follow up threshold: all; and Auto select best cutoff and user selected probe set were selected to generate these figures); (**b**) The correlation of CB_2_ expression with clinical factors of HCC patients; (**c**) Three siRNAs targeting CB_2_ were synthesized and introduced into Hep3B and HepG2 cells. The mRNA expression of CB_2_ was detected by qRT-PCR. **d** CB_2_ was knocked down by using RNAi technology and western blot was used to detect the interference efficiency on protein level; (**e**) CCK8 was used to detect cell proliferation of HCC cells in NC (negative control), CB_2_-KD and MDA19 + CB_2_-KD groups. NC: HCC cells were transfected with siNC (50nM); CB_2_-KD: HCC cells were transfected with CB_2_ siRNA (50nM); MDA19 + CB_2_-KD: HCC cells were transfected with CB2 siRNA (50nM) and treated with MDA19 (30μM for Hep3B and 40μM for HepG2). All experiments were performed at 3 times. *P < 0.05

MDA19 was reported as an agonist for CB_2_, so we further investigated the function of CB_2_ in HCC. The Kaplan-Meier plotter (http://kmplot.com/analysis/) is an online analyzing tool for evaluating the survival effect of a gene or combination of genes in breast, ovarian, lung, liver and gastric cancers [19]. The Kaplan-Meier survival curves for HCC patients with low and high CB_2_ expression were shown in Fig. 4a, which illustrated better prognosis for those HCC patients with high CB_2_ expression. As shown in Fig. 4b, the expression of CB_2_ was significantly correlated with gender, clinical stages and race of HCC patients (*P* < 0.05). Taken together, these observations suggested that CB_2_ expression was associated with a better prognosis of HCC patients.

### CB_2_ knockdown reversed the inhibition of HCC cell proliferation induced by MDA19

To comprehensively evaluate the effect of the interaction between MDA19 and CB_2_ on HCC cells, we used RNAi technology to reduce the expression of CB_2_ in HCC cells, and combined it with MDA19 treatment. As shown in Fig. 4c, compared to NC group, the mRNA expression of CB_2_ was significantly inhibited by siRNAs in both Hep3B and HepG2 cells. siRNA1 was used for the following experiments. The inference effects were also validated on protein level using western blot assay (Fig. 4d). Figure 4e indicated that CB_2_ knockdown significantly promoted cell proliferation compared with NC group. Moreover, CB_2_ knockdown reversed the inhibition of HCC cells by MDA19, which proved that the anti-tumor activity of MDA19 was mediated by activating CB_2_ (Fig. 4e). These data suggested that CB_2_ functions as a negative regulator in HCC proliferation and the anti-tumor activity of MDA19 was dependent on CB_2_ expression.

### CB_2_ knockdown inhibited cell apoptosis of HCC

**Fig. 5:**
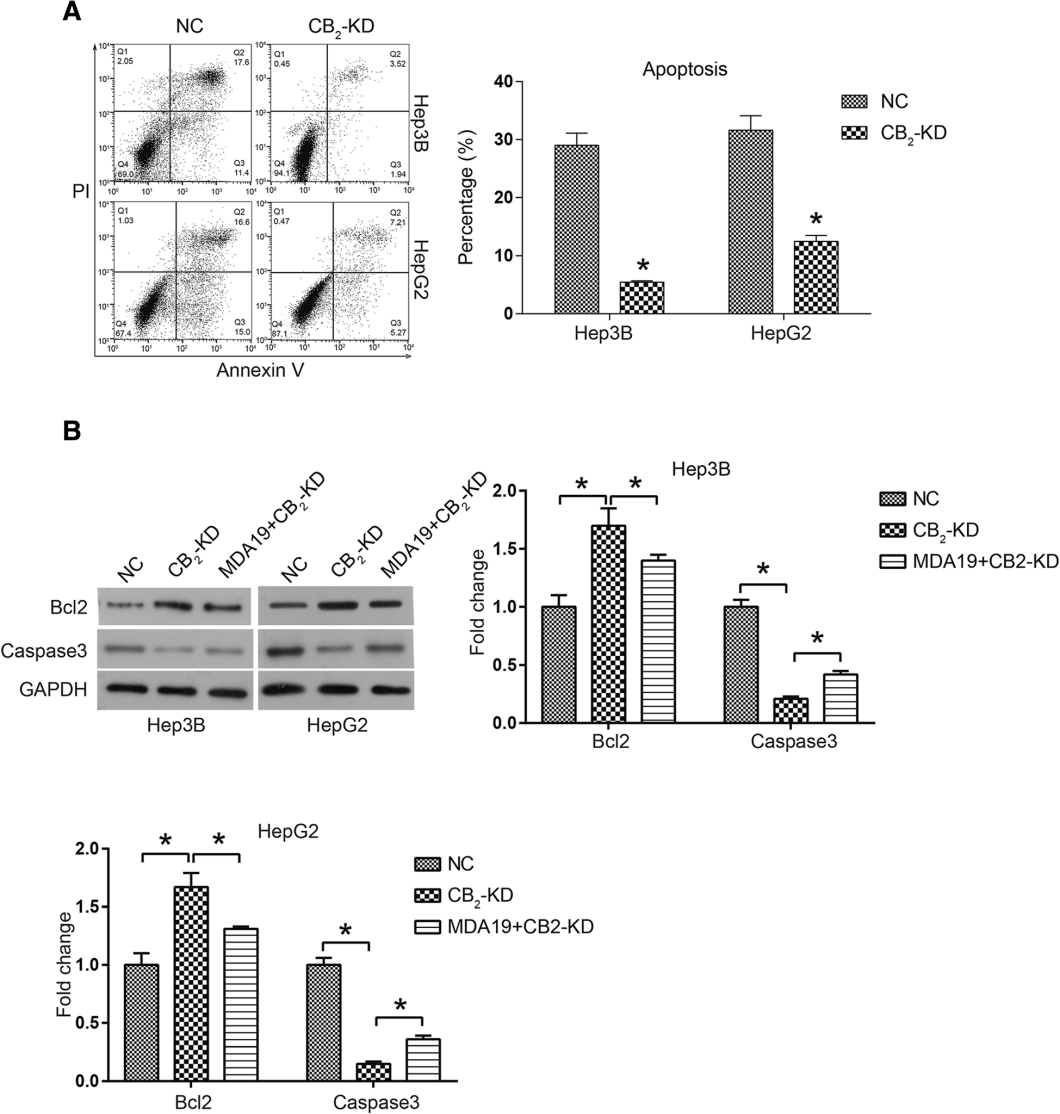
CB_2_ knockdown inhibited cell apoptosis of HCC. NC: HCC cells were transfected with siNC (50nM) and incubated for 48h; CB_2_-KD: HCC cells were transfected with CB_2_ siRNA (50nM) and incubated for 48 h; MDA19 + CB_2_-KD: HCC cells were transfected with CB2 siRNA (50nM) and treated with MDA19 (30 μM for Hep3B and 40μM for HepG2) for 48 h. **a** Cell apoptosis of HCC cells was detected by a PI-AnnexinV-FITC assay and flow cytometry; The data were analyzed using FlowJo software.; (**b**) The expression of apoptosis related proteins Bcl2 and Caspase3 was detected by western blot and analyzed by Image J software. All experiments were performed at 3 times. *P < 0.05

Cell apoptosis analysis was used to determine the effect of CB_2_ knockdown on HCC cell survival. HCC cells were starved in serum-free medium for 24 h to induce cell apoptosis. As shown in Fig. 5a, compared with NC group, CB_2_ knockdown significantly reduced apoptosis percentage in HCC cells (*P* < 0.05). Mitochondrial apoptosis pathway was also examined by western blot assay. As expected, the expression of anti-apoptotic =protein Bcl-2 was up-regulated in CB_2_-KD group, while pro-apoptotic proteins Caspase3 were down-regulated (*P* < 0.05, Fig. 5b). Furthermore, we found that CB_2_-KD could reverse the effects of MDA19 on the expression of apoptosis-related protein expression (Fig. 5b). These data suggested that CB_2_ knockdown inhibited HCC cell apoptosis through inactivation of mitochondrial-dependent apoptosis pathway and the pro-apoptotic effects of MDA19 on HCC cells might be mediated by CB_2_.

### CB_2_ knockdown promoted cell mobility in HCC and activated AKT signaling pathway

**Fig. 6:**
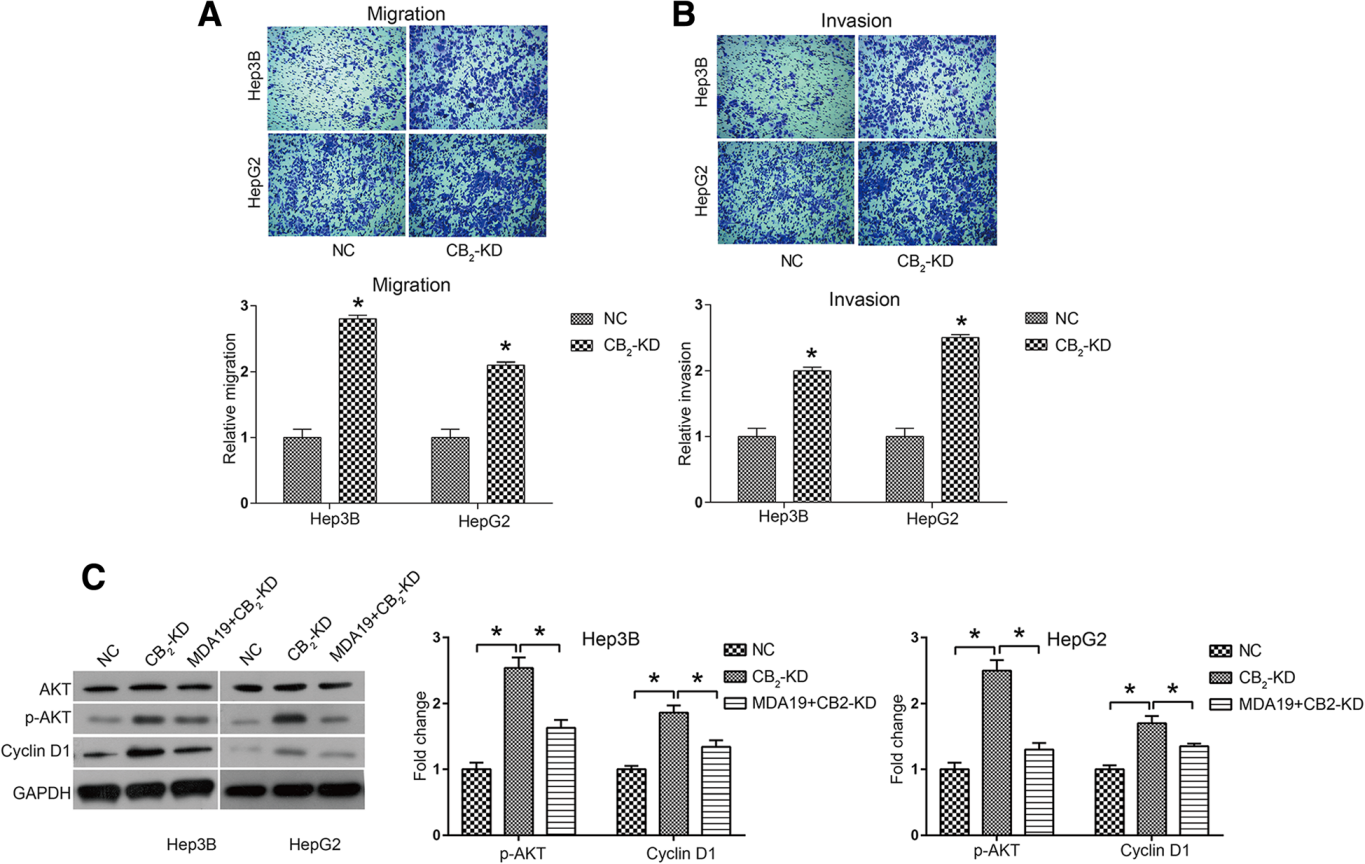
CB_2_ knockdown promoted HCC cell mobility and activated AKT signaling pathway NC: HCC cells were transfected with siNC (50nM); CB_2_-KD: HCC cells were transfected with CB_2_ siRNA (50nM); MDA19 + CB_2_-KD: HCC cells were transfected with CB2 siRNA (50nM) and treated with MDA19 (30μM for Hep3B and 40μM for HepG2) for 48 h. **a** Cell migration and (**b**) cell invasion were detected by transwell assay. **c** AKT signaling pathway components, including AKT, p-AKT, CDK4, CDK6 and Cyclin D1, were detected by western blot and analyzed by Image J software. All experiments were performed at 3 times. *P < 0.05

The effect of CB_2_ knockdown on HCC cell mobility was determined by a transwell assay. As shown in Fig. 6a, CB_2_ knockdown significantly promoted cell migration in Hep3B and HepG2 cells. Figure 6b revealed that CB_2_ knockdown also promoted Hep3B cell invasion by 2 fold and HepG2 by 2.5 fold. Thus, it was suggested that CB_2_ knockdown increased the mobility of HCC cells.

We further investigated whether CB_2_ was also involved in the regulation of AKT signaling pathway. As shown in Fig. 6c, it was suggested that p-AKT and Cyclin D1 were both up-regulated by CB_2_ knockdown. Furthermore, CB_2_-KD reversed the inhibitory effect of MDA19 on AKT signaling pathway in both Hep3B and HepG2 cells (Fig. 6c). These data indicated that CB_2_ knockdown could activate AKT signaling pathway and MDA19 functioned as a negative regulator of AKT pathway through interaction with CB_2_.

## Discussion

Agonists selective for cannabinoid receptor 2 (CB_2_) are shown to inhibit tumor growth through inducing PI3K/AKT signaling, MAPK/ERK signaling and so on [20–22]. For example JWH-015 treatment significantly inhibits tumor growth and metastasis of 4 T1 cells in vivo [20]. Cannabinoids inhibit glioma cell invasion by down-regulating matrix metalloproteinase-2 expression [21]. In this study, we demonstrated that MDA19, a small-molecule CB_2_ agonist, exerted an anti-tumor activity in HCC.

Cell proliferation analysis showed that MDA19 treatment inhibited cell viability in a dose- and time-dependent manner in HCC cells. IC50 values were 56.69 μM for Hep3B cells and 71.13 μM for HepG2 cells. Apoptosis analysis showed that MDA19 treatment significantly increased the proportion of apoptosis in HCC cells, and the induction of apoptosis was mediated by activation of mitochondrial-dependent apoptosis pathway, including increased Bax and Caspase3 and decreased Bcl2. When mitochondrial-dependent apoptosis pathway is activated, increased Bax moves to the mitochondrial outer membrane and multimerizes, forming membrane channels that stimulate mitochondria to release cytochrome C (Cyt C) [23, 24]. Cyt C triggers cell apoptosis through Caspase9/3 cascade reaction [23, 24]. Bcl2 exerts anti-apoptosis effect by antagonizing Bax in this process [23, 24]. In addition, some CB_2_ agonists have also been reported to exert a suppressive effect on tumor metastasis [12–14]. Here by using transwell assay we reported that MDA19 significantly inhibited HCC cell migration and invasion.

Several mechanisms of the anti-tumor activity of CB_2_ agonists have been reported, including PI3K/AKT inhibition [25], modification of metalloproteinases [21], induction of reactive oxygen species [26], MAPK modulation [20] and so on. Here, our data showed that MDA19 treatment led to inactivation of AKT signaling pathway in HCC cells. It is widely held that AKT signaling pathway plays a vital role in maintaining cell proliferation, survival and cell cycle progression [27, 28]. And increasing studies demonstrate that inactivation of AKT signaling pathway inhibits tumor growth and enhances the sensitivity of tumor cells to chemotherapy drugs such as cisplatin and temozolomide [29–31], suggesting that inhibition of AKT pathway may be a promising strategy for HCC treatment.

Emerging evidences suggest that the cannabinoid receptor CB_2_ can be an anti-tumor target in several types of cancers [32–34]. For example, Cianchi et al. report that CB_2_ activation induces apoptosis through tumor necrosis factor alpha-mediated ceramide de novo synthesis in colon cancer cells [33]. Moreover, Khan et al. report that CB_2_ is involved in inducing cell cycle arrest and apoptosis in renal cell carcinoma [35]. We would like to know whether MDA19, an agonist of CB_2_, exerts its anti-tumor activity by interacting with CB_2_. We investigated the relationship between CB_2_ expression and the survival rate of HCC patients on Kaplan-Meier plotter (http://kmplot.com/analysis/), suggesting that the survival rate of HCC patients with high expression of CB_2_ was significantly higher than that of patients with low expression of CB_2_. Moreover, cell viability of CB_2_ silenced HCC cells was significantly higher than that of control group. These results indicated that CB_2_ exerted an anti-tumor function in HCC. In addition, we examined the effect of CB_2_ knockdown on proliferation, apoptosis, invasion, and migration in HCC cells. The results showed that knockdown of CB_2_ could reverse MDA19-induced growth inhibition in HCC cells. Mechanism studies suggested that CB_2_ knockdown also inhibited mitochondrial-dependent apoptosis pathway and activated AKT signaling pathway. These findings suggested that CB_2_ played an anti-tumor role in HCC, which is consistent with the previous studies.

## Conclusions

In conclusion, these data suggest that MDA-19 exerts an anti-tumor activity at least partly through inhibiting AKT signaling pathway in HCC. CB_2_ functions as a tumor suppressor gene in HCC, and the anti-tumor activity of MDA19 depends on its binding to CB_2_ to activate it.

## Reviewers’ comments

### Reviewer’s report 1 Tito Cali

Reviewer comments:

Reviewer1: MDA19, a novel CB2 agonist, inhibits hepatocellular carcinoma through inactivation of AKT signalling pathway This manuscript investigates the inhibitory role of the cannabinoid receptor 2 (CB2) agonist MDA19 in cancer progression, by focusing on hepatocellular carcinoma (HCC) cell lines (Hep3B and HepG2). Rao, M. and colleagues demonstrate that MDA19 treatment inhibited HCC cell proliferation (dose- and time dependently), migration and invasion. They further show that growth inhibition might be mediated by cell apoptosis and activation of mitochondrial dependent pathway. Mechanistically, MDA19 is proposed to perform its action through inactivation of the AKT pathway. The MDA19-induced phenotype changes observed in HCC cells, including activation of the AKT signalling pathway, could be reversed by CB2 knockdown. Lastly, and interestingly, the authors also found a direct and positive correlation between CB2 expression and the survival of HCC patients as well as association with clinical factors such as gender, clinical stages and race. The authors conclude that MDA-19 exerts an anti-tumor activity through inactivation of AKT signalling pathway in HCC, therefore MDA-19 treatment may be a promising strategy for HCC therapy. As a general comment, with few exceptions, the overall manuscript is of immediate understanding. Concepts and descriptions are explained in a satisfactory manner and the Results section, although sometimes too succinct, is somehow in line with the experimental data. The Discussion is pertinent with the Results and the topic, and takes into account the recent literature in the field. The experimental data provided seem to be sufficient to support some of the conclusions drawn by the authors, nevertheless, there are some major points to be addressed. Major Points.

1. The experiments shown in Fig. 1a are performed at 24 h (see methods section), under these conditions the authors state that “The significant cytotoxic effect of MDA19 was starting at concentration of 10 μM for both HCC cell lines…. 30 μM for Hep3B cells and 40 μM for HepG2 cells were used for the following experiments”. At 24 h, 30 μM for Hep3B cells and 40 μM for HepG2 are sufficient to induce a strong cytotoxic effect as shown in Fig. 1a, nevertheless, this is not found in Fig. 1b under the same experimental conditions where the effect at 24 h with the above-mentioned concentrations of MDA19 is not statistically significant. How do the authors explain this apparent discrepancy?

2. Concerning the figure legends, they should all be carefully revised since many details are lacking and the experiments are not described in a satisfactory manner. Please include the important information such as the time of treatment, concentrations and also briefly describe the experiment since the results section is sometimes too descriptive or not enough exhaustive.

3. The dose of MDA looks quite high (30–40 micromolar). This is important when considering the compound as promising for therapy. The authors should perform additional experiments to exclude extra side effects by looking at signalling pathways and/or cellular processes supposed not to be affected by the compound. MDA19 is a selective agonist of the human peripheral cannabinoid (CB2) receptor, the EC50 value for activation is reported to be 63.4 nM, well below the micromolar concentration used in this study. I think that this cast doubts about the specificity of the observed phenotypes and the therapeutic potential of the compound.

4. Is the compound cell permeant? The molecule is lipophilic so I think that the authors should perform experiments with selective CB2 antagonist such as AM630 to assess the effectiveness and the specificity of this compound for the observed phenotypes.

5. For the CB2 knockdown, the results should be replicated by using at least three independent specific siRNA and a scramble siRNA as further control. Additionally, can the authors abolish (or at least strongly reduce) the effect of MDA19 treatment in the CB2-KD cells? The western blots shown in Figs. 5b and 6c should be performed by adding a CB2-KD treated group as done for the experiments shown in Fig. 4d.

6. On the same line, HCC cells also express CB1 receptor, do the authors observe the same effect by downregulating the CB1 receptor in the presence and/or in the absence of MDA19?

7. Along this line, can the authors phenocopy the effect of CB2 knockdown by treatment of the cells with specific CB2 antagonists (e.g., AM630 or others)?

8. In the Western blot shown in Fig. 2b, the intensity of the Bcl2 and Caspase3 bands in the control group is much higher than that reported in Fig. 5b for the same condition. This could be due to the fact that different exposure times are used. To increase the clarity of the phenotype and to better understand the relative levels of the markers taken into account between the two cell lines the western blots and the quantifications should be done by loading the Hep3B and the HepG2 lysates on the same membrane. This is true for Figs. 2, 3, 4 and 5. This is also due to the fact that Hep3B and HepG2 cells are also quite different in terms of aggressiveness, metabolic and growth rate, ability to form tumors in mice, presence absence of p53 mutations etc.

9. Last but not least there is an important concern to be addressed: the authors show an effect on mitochondrial targets (but not all of them) and AKT pathway, but there is an important control that should be performed, i.e., the lack of effect on unrelated pathways. For example, are markers of the extrinsic pathway unchanged? is there any ER stress ongoing? Are the markers (phosphorylation status) of pathways other than AKT unchanged? To state that the AKT pathway and/or that the mitochondria-mediated apoptosis are involved the authors should show that other signalling pathways are indeed unchanged. In conclusion, the potential of MDA19 in cancer therapy is interesting but some concerns regarding the experimental data should be carefully edited before to consider the paper of interest for publication in Biology Direct.

10. Page 7 end of line 13 Cone please correct with clone. Page 7 middle of line 10 “treatment with MDA inhibited Hep3B and HepG2,,,” is not clear what is inhibited, growth? Colony formation?

Author’s response:

*1. We are very sorry that we made a mistake on the description of Dose-dependent assay method. The treatment time of MDA19 was 48 h, rather than 24 h.*

*2. The figure legends have been revised and added the necessary information such as experiment description.*

*3. We have read the article “Design and Synthesis of a Novel Series of N-Alkyl Isatin Acylhydrazone Derivatives that Act as Selective Cannabinoid Receptor 2 Agonists for the Treatment of Neuropathic Pain” reported by Diaz et al. MDA19 exhibited a low EC50 value for activation (63.4 nM) for CB2 receptor. It is very different from our study. As the reported experiments were based on Chinese hamster ovarian cells selectively expressing the human CB2 receptor, we believe that this difference may be due to the high complexity and specificity of tumor microenvironment. The killing effect of MDA19 on tumor cells is affected by the signaling pathways of various mutations in tumor cells in addition to the affinity of the receptor. In a recent study reported by Liu et al., the IC50 of MDA19 was 20 μM for human osteosarcoma cells.*

*4. Your advice is very useful and we will performed the related experiments in the future. In this study we only demonstrated that CB2 knockdown could reverse the growth inhibition induced by MDA19.*

*5. We designed three CB2 siRNAs and evaluated the inference efficiencies by qRT-PCR. Because MDA19 could affect the RNA inference, the treatment of “MDA19 + CB2-KD” was as follow: HCC cells were pre-transfected with CB2 siRNA and then treated with MDA19 (30 μM for Hep3B and 40 μM for HepG2). The “MDA19 + CB2-KD” has been added in Fig.* *5* *b and* *6* *c.*

*6. Your advice is very constructive. However, due to limitation of time and experimental condition, we will perform the related evaluation in the future.*

*7. Your advice is very useful. However, due to limitation of time and experimental condition, we will perform the related evaluation in the future.*

*8. The western blots and the quantifications have be done by loading the Hep3B and the HepG2 lysates on the same membrane in Fig.* *2* *. However, for Fig.* *5* *, we failed to take them in one membrane. But we assure that they were exposed for the same time.*

*9. Your advice is very constructive. However, due to the experimental time and conditions, we will make further studies in the future. Here for the accurate description of our results, we declare that the anti-tumor activity of MDA19 is at least partly through AKT signaling pathway.*

*10. We have corrected the two mistakes in the manuscript.*

### Reviewer’s report 2 Mohamed Naguib

Reviewer comments:

The manuscript by Rao, et al. described the anti-tumor effect of novel specific CB2 agonist MDA19 in HCC cell lines (Hep3B and HepG2). They elucidated the potential intracellular mechanism in this process. Overall, this study added some significant novel points in this field of research. Some minor issues:

1. While the manuscript is basically drafted well in a logical way, the quality of the manuscript could be improved if the authors have someone proofread the full paper.

2. Page 3, LM:35–38: There is no evidence that cannabinoids derived from marijuana has any potential therapeutic effect in cancer or any other medical condition. References 7 and 8 cited by the authors are old reviews and the authors should cite definite clinical studies. The authors should review this article: “Medical Use of Marijuana: Truth in Evidence. Anesth Analg 121 (5):1124-1127”.

3. The authors need to provide the necessary information about vendors for the key reagents (e.g., MDA19) and antibodies.

4. The authors should provide more details on statistical analyses (normality test, n, t value or F value, etc.)

5. The authors need to provide more details on the Kaplan-Meier curve (Fig. 4a). What is the origin of the data presented in this figure? The legend provided by the authors is inadequate.

6. The authors should have considered studying a separate group with specific CB2 agonist, at least, the authors need comment the CB2 selectivity of MDA19.

Author’s response:

*1. We have checked throughout the manuscript and tried our best to improve the manuscript quality.*

*2. We have read the review about medical use of marijuana and deleted the wrong description of therapeutic effect of cannabinoids derived from marijuana in cancer.*

*3. The necessary information about vendors for MDA19, antibodies et al. has been added in the manuscript.*

*4. We have added the necessary information in the statistical analysis.*

*5. The detailed information for Kaplan-Meier curve (Fig.* *4* *a) and the data origin has been added in the manuscript.*

*6. We have commented the CB2 selectivity of MDA19 in the manuscript. “MDA19 displayed 4-fold-higher affinity at the human CB2 than at the human CB, receptor and nearly 70-fold-higher affinity at the rat CB2 than at the rat CB1 receptor”.*

### Reviewer’s report 3 Bo Chen

Reviewer comments:

This manuscript demonstrates that MDA19 acts as a CB2 agonist to inhibit cell proliferation, migration and invasion through AKT pathway in HCC cell lines. This is an interesting report, but some improvements needs to be done to validate the findings.

1. Fig. 1 Statistics on the number of clones may have errors, please recount.

2. Fig. 5, why apoptosis percentage in negative control group was so high? Was cells in NC group treated with starving? Please improve it in the methods.

3. The author should add more details of the previous studies of anti-tumor function of CB2 in discussion and compare with the present findings.

4. There are some minor grammatical errors in the manuscript. Please check and correct.

Author’s response:

*1. The number of clone in* Fig. 1 *has been re-counted.*

*2. The cells also went through a starve process prior to apoptosis analysis. We have added this in the methods.*

*3. We have added the the previous studies of anti-tumor function of CB2 in discussion and compare with the present findings.*

*4. We have checked throughout the manuscript and correct the grammatical mistakes.*
